# Geostatistical analysis of disease data: visualization and propagation of spatial uncertainty in cancer mortality risk using Poisson kriging and p-field simulation

**DOI:** 10.1186/1476-072X-5-7

**Published:** 2006-02-09

**Authors:** Pierre Goovaerts

**Affiliations:** 1 BioMedware, Inc., Ann Arbor, MI, USA

## Abstract

**Background:**

Smoothing methods have been developed to improve the reliability of risk cancer estimates from sparsely populated geographical entities. Filtering local details of the spatial variation of the risk leads however to the detection of larger clusters of low or high cancer risk while most spatial outliers are filtered out. Static maps of risk estimates and the associated prediction variance also fail to depict the uncertainty attached to the spatial distribution of risk values and does not allow its propagation through local cluster analysis. This paper presents a geostatistical methodology to generate multiple realizations of the spatial distribution of risk values. These maps are then fed into spatial operators, such as in local cluster analysis, allowing one to assess how risk spatial uncertainty translates into uncertainty about the location of spatial clusters and outliers. This novel approach is applied to age-adjusted breast and pancreatic cancer mortality rates recorded for white females in 295 US counties of the Northeast (1970–1994). A public-domain executable with example datasets is provided.

**Results:**

Geostatistical simulation generates risk maps that are more variable than the smooth risk map estimated by Poisson kriging and reproduce better the spatial pattern captured by the risk semivariogram model. Local cluster analysis of the set of simulated risk maps leads to a clear visualization of the lower reliability of the classification obtained for pancreatic cancer versus breast cancer: only a few counties in the large cluster of low risk detected in West Virginia and Southern Pennsylvania are significant over 90% of all simulations. On the other hand, the cluster of high breast cancer mortality in Niagara county, detected after application of Poisson kriging, appears on 60% of simulated risk maps. Sensitivity analysis shows that 500 realizations are needed to achieve a stable classification for pancreatic cancer, while convergence is reached for less than 300 realizations for breast cancer.

**Conclusion:**

The approach presented in this paper enables researchers to generate a set of simulated risk maps that are more realistic than a single map of smoothed mortality rates and allow the propagation of cancer risk uncertainty through local cluster analysis. Coupled with visualization and querying capabilities of geographical information systems, animated display of realizations can highlight areas that depart consistently from the general behavior observed across the region, guiding further investigation and control activities.

## Background

Cancer mortality maps are used by public health officials to identify areas of excess and to guide surveillance and control activities. Quality of decision-making thus relies on an accurate quantification of risks from observed rates which can be very unreliable when computed from sparsely populated geographical entities or for diseases with a low frequency of occurrence [1]. Smoothing methods have been developed to improve the reliability of these estimates by borrowing information from neighboring entities [2,3]. These methods range from simple deterministic techniques (i.e. head banging method [1]) to sophisticated full Bayesian models [4,5]. Empirical Bayes smoothers [6,7] and Poisson kriging [8] provide model-based approaches with intermediate difficulty in terms of implementation and computer requirements. Although simulation studies [7,8] have demonstrated the benefit of smoothing methods for risk prediction, some uncertainty will always be associated with the estimated risk. In Poisson kriging the uncertainty about the risk within a given geographical entity is modeled by computing a minimum error variance (kriging) estimate of the risk and the associated error variance, which can then be combined to derive a Gaussian-type confidence interval. Full Bayes models go one step further and yields the full posterior distribution of the risk while accounting for the uncertainty in the parameters of the model.

Most studies do not make use of the uncertainty measure provided by smoothing methods, and only the map of smoothed rates is reported and used in the analysis. This is unfortunate since all rate smoothers, including Poisson kriging, cause the loss of local details of the spatial variation of the risk. This smoothing potentially affects the subsequent analysis, leading for example to the detection of larger aggregates of low or high cancer risk in local cluster analysis while most spatial outliers are filtered out [9]. To depict the uncertainty attached to risk maps, some authors recommend mapping the 95^th ^percentile range of the posterior distribution of risk values or the probability that the risk in each entity exceeds a specific threshold of interest [4,10,11]. Richardson *et al. *[12] proposed to use this probability of exceedence to decide whether an area should be classified as having an excess risk of cancer. They discussed different decision rules or loss functions which represent weighted trade-offs between false-positive results (i.e. declaring an area as having elevated risk when in fact its underlying true risk equals the background level) and false-negative results (i.e. declaring an area to be in the background when in facts its underlying risk is elevated).

A major weakness of the uncertainty measures reported in today's health science literature is that they are area-specific, that is they inform on the uncertainty prevailing over a single geographical entity at a time. Except if the risk values are spatially independent, the probabilities of excess of a specific threshold computed for several entities do not provide a measure of uncertainty about whether these entities jointly exceed that threshold. In addition to a measure of "local" uncertainty, one thus needs to assess the "spatial" uncertainty, that is the uncertainty attached to the spatial distribution of risk values across the study area. The quantification of spatial uncertainty is particularly important for cluster detection, since the focus is on risk values for a group of geographical entities considered simultaneously. This information is not conveyed by a statistic map of the estimated risk and the associated prediction variance.

Spatial uncertainty modeling has been one of the most vibrant areas of research in geostatistics for the last 2 decades [13-15]. Applications, such as modeling the migration of pollutants in the subsurface environment, require measures of multiple-point uncertainty, such as the probability of occurrence of a string of high or low permeability values that may represent a flow path or flow barrier [16]. These joint probabilities are assessed numerically from a set of realizations of the spatial distribution of attribute values over the locations of interest. In other words, the spatial uncertainty is modeled through the generation of a set of equally-probable simulated maps, each consistent with the information available, such as histogram or a spatial correlation function. Stochastic simulation allows generation of maps that reproduce the spatial variability of the data without smoothing effect [17]. The set of simulated maps can also be used to propagate the uncertainty through spatial operators or transfer functions. For example, the set of simulated permeability maps can be fed into a flow simulator, yielding a distribution of response values, such as travel times to the water table [18].

From the user's perspective, it is important to be able to visualize the uncertainty in the spatial model of risk values. Although stochastic simulation offers a way to generate a large number of potential scenarios, the burden of manually scrolling through hundreds of different maps will test the patience of most users and be little informative. The information contained in the set of simulated maps is thus often summarized through a **static display **of probabilities of exceeding particular threshold or some measures of the spread of the posterior distribution [13]. By doing so, one however fails to depict the uncertainty about spatial features and essentially maps the area-specific measures of uncertainty provided by kriging and other smoothing methods. To depict visually the spatial uncertainty, several authors [19,20] have developed algorithms that show the realizations one at a time in rapid succession, like the frames of an animated cartoon, thereby eliminating the burden of manually displaying each simulated map. Like an animated cartoon, successive realizations must be similar enough to allow the eye to catch gradual changes. Such a similarity can be achieved by ranking the realizations appropriately or by using a simulation algorithm, called p-field simulation [21-23], that generates realizations that are incrementally different. The **animated display **of realizations allows one to distinguish areas that remain stable over all realizations (low uncertainty) from those where large fluctuations occur between realizations (high uncertainty).

This paper presents the first application of geostatistical simulation to model the spatial uncertainty attached to cancer risk values. This approach combines Poisson kriging and p-field simulation to generate multiple realizations of the spatial distribution of cancer risk values. These simulated maps are then fed into a local cluster analysis, allowing one to assess how uncertainty about the spatial distribution of risk values translates into uncertainty about the location of spatial clusters and outliers in cancer risks. The simulation approach is also used to generate a series of realizations that are incrementally different and animations are created using the Space-Time Information System (STIS) technology [24]. A public-domain executable for generating multiple risk maps, along with example datasets, is provided. This novel methodology is applied to the modeling, visualization and propagation of the spatial uncertainty of age-adjusted breast and pancreatic cancer mortality risks for white females in 295 US counties of the Northeast (1970–1994).

## Methods

### Data

**Figure 1:**
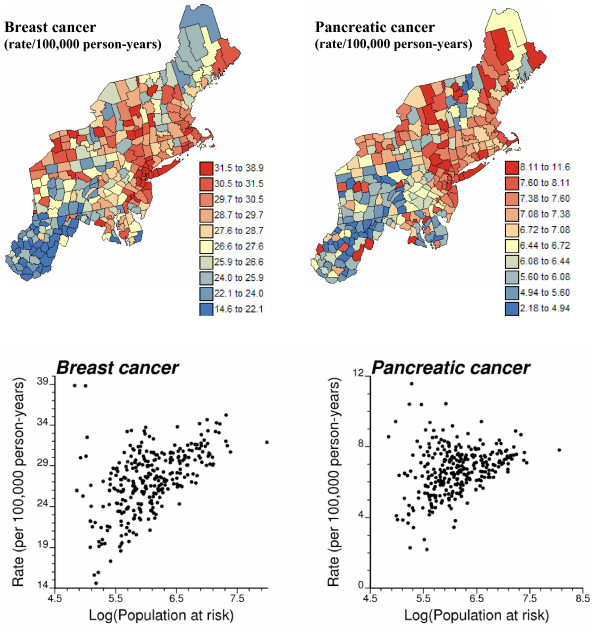
**Geographic distribution of age-adjusted breast and pancreatic cancer mortality rates for white females**. For the two top maps, the fill color in each county represents the age-adjusted mortality rates per 100,000 person-years recorded over the period 1970–1994 for white females. The class boundaries correspond to deciles of the histogram of rates. The scattergrams illustrate the larger variability in rates observed for counties with small populations.

The methodology for modeling and propagating the uncertainty about the spatial distribution of health data will be illustrated using directly age-adjusted mortality rates for a frequent (i.e. breast) and less frequent (i.e. pancreatic) cancer. These data are part of the Atlas of Cancer Mortality in the United States [25] and were downloaded from . The rates were adjusted using the 1970 population pyramid. The analysis focuses on white female rates recorded over the 1970–1994 period for 295 counties of 12 New England States. Figure 1 (top graphs) shows the spatial distribution of age-adjusted mortality rates per 100,000 person-years. Following the recommendations of several studies on map color schemes [26,27], a double-ended color scheme with 10 equally-weighted classes (i.e. boundaries correspond to deciles of the histogram) was used: a gradient of red is used for rates higher than the median, while a gradient of blue is used for lower rates. The bottom scattergrams indicate that extreme high or low rates are typically recorded for sparsely populated counties (small number problem). For both cancers, the population at risk was computed as: 100,000 × the total number of deaths over the 1970–1994 period divided by the age-adjusted cancer mortality rate; both datasets are available on NCI website. The population-weighted average of the age-adjusted mortality rates is 30.14 per 100,000 person-years for breast cancer and 7.25 per 100,000 person-years for pancreatic cancer.

### Poisson kriging

The geostatistical methodology for the estimation of risk values from empirical frequencies, and its performance relative to common smoothers, is described in details in Goovaerts [8]. This section briefly reviews its most salient features. For a given number *N *of entities (e.g. counties, states, electoral ward), denote the observed mortality rates as *z*(**u**_*α*_) = *d*(**u**_*α*_)/*n*(**u**_*α*_), where *d*(**u**_*α*_) is the number of recorded mortality cases and *n*(**u**_*α*_) is the size of the population at risk. These entities are referenced geographically by their centroids (or seats) with the vector of spatial coordinates **u**_*α *_= (x_*α*_,y_*α*_). The disease count *d*(**u**_*α*_) is interpreted as a realization of a random variable *D*(**u**_*α*_) that follows a Poisson distribution with one parameter (expected number of counts) that is the product of the population size *n*(**u**_*α*_) by the local risk *R*(**u**_*α*_).

The risk estimate over a given entity with centroid **u**_*α*_, and the attached prediction variance, are computed from *K *neighboring observed rates as:





where *C*_R_(**u**_i_-**u**_*α*_) is the covariance between the rate measured at **u**_i _and the risk estimated at **u**_*α *_; the spatial proximity is here defined in terms of Euclidian distance between the geographical centroids of the corresponding counties. The kriging weights *λ*_*i*_(**u**_*α*_) are the solution of the following system of linear equations, known as the "Poisson Kriging" (PK) system [28,29]:



where *δ*_ij _= 1 if **u**_i _= **u**_j _and 0 otherwise, and m* is the population-weighted mean of the rates. The term *μ*(**u**_*α*_) is a Lagrange parameter that results from the minimization of the estimation variance subject to the unbiasedness constraint on the estimator. The addition of an "error variance" term, m*/*n*(**u**_i_), for a zero distance accounts for variability arising from population size, leading to smaller weights for less reliable data (i.e. measured over smaller populations).

The computation of kriging weights and kriging variance requires knowledge of the covariance of the unknown risk, *C*_*R*_(**h**), or equivalently its semivariogram *γ*_*R*_(**h**) = *C*_*R*_(0)-*C*_*R*_(**h**). Following Monestiez *et al. *[28,29] the semivariogram of the risk is estimated as:



where N(**h**) is the number of pairs of county centroids separated by a vector **h**. The different pairs [*z*(**u**_*α*_)-*z*(**u**_*α*_+**h**)] are weighted by the corresponding population sizes to homogenize their variance. A permissible model, *γ*_*R*_(**h**), is then fitted to the experimental semivariogram, i.e. using weighted least-square regression [30] in this paper.

### Modeling local and spatial uncertainty

The uncertainty about the cancer mortality risk prevailing within the county with centroid **u**_*α *_can be modeled using the conditional cumulative distribution function (ccdf) of the risk variable *R*(**u**_*α*_) defined as:



where *G*(.) is the cumulative distribution function of the standard normal random variable. The notation "|(*K*)" expresses conditioning to the local information, say, *K *neighboring observed rates. The function (5) gives the probability that the unknown risk is no greater than any given threshold *r*. It is modeled as a Gaussian distribution with the mean and variance corresponding to the Poisson kriging estimate and variance. Measures of the spread of the distribution (i.e. variance or interquartile range), as well as the probability of exceeding any given threshold, are easily computed and mapped [[13], p. 358–361].

Instead of a unique set of smooth risk estimates {(**u**_*α*_), *α *= 1,...,*N*}, stochastic simulation aims to generate a set of *L *equally-probable realizations of the spatial distribution of risk values, {*r*^(l)^(**u**_*α*_), *α *=1,...,*N*; *l *= 1,...,*L*}, each consistent with the spatial pattern of the risk as modeled using the function *γ*_*R*_(**h**). Simulation of spatial phenomena can be accomplished using a growing variety of techniques that differ in the underlying random function model, the amount and type of information that can be accounted for, and the computer requirements [13]. The main difficulty for the current application is that there is no measured risk data; only a semivariogram indirectly computed from observed rates according to expression (4) is available. The lack of target histogram is not an issue for the p-field simulation approach [21-23]. The basic idea is to generate a realization {*r*^(l)^(**u**_*α*_), *α *= 1,...,*N*} through the sampling of the set of ccdfs by a set of spatially correlated probability values {*p*^(l)^(**u**_*α*_), *α *= 1,...,*N*}, known as a probability field or p-field. Since the probability distributions are Gaussian (Equation 5), each risk value is simulated as:

*r*^(*l*)^(**u**_*α*_) = *F*^-1^(**u**_*α*_; *p*^(*l*)^(**u**_*α*_) | (*K*)) = (**u**_*α*_) + *σ*_*PK*_(**u**_*α*_)*y*^(*l*)^(**u**_*α*_)     (Equation 6)

where *y*^(*l*)^(**u**_*α*_) is the quantile of the standard normal distribution corresponding to the cumulative probability *p*^(*l*)^(**u**_*α*_).

#### Generating the probability field

The *L *sets of random deviates or normal scores, {*y*^(*l*)^(**u**_*α*_), *α *= 1,...*N*}, are generated using non-conditional sequential Gaussian simulation which proceeds as follows (see [13], p. 380 for more details):

1. Define a random path (i.e. using a random number generator) visiting each county centroid **u**_*α *_only once.

2. At each location **u**_*α *_determine the mean and variance of the Gaussian probability distribution of *y*-values as:





where *y*^(l)^(**u**_i_) are normal scores simulated at locations previously visited along the random path and located within a search radius from **u**_*α*_, and *C*(**u**_i_-**u**_*α*_) is the covariance function of the normal score variable Y for the separation vector **h**_i*α *_= **u**_i_-**u**_*α *_. In this paper, *C*(**h**) was identified to the covariance function of the risk after rescaling to a unit sill, i.e. *C*(**h**) = 1-*γ*_*R*_(**h**)/*C*_*R*_(0). The *λ*_i _are kriging weights obtained by solving the following system of linear equations (simple kriging, SK):



3. Draw a simulated value from the Gaussian ccdf and add it to the data set. In other words, the simulated value at **u**_*α *_is *y*^(l)^(**u**_*α*_) =  (**u**_*α*_)+*σ*_*SK*_(**u**_*α*_) × *G*^-1 ^[*p*^(l)^], where *p*^(l) ^is a random number between 0 and 1.

4. Proceed to the next location along the random path, and repeat the two previous steps.

5. Loop until all *N *locations (i.e. N = 295 here) are simulated.

The procedure is repeated using a different random path and set of random numbers to generate another realization. Note that the mean and variance of the set of simulated normal scores can sometimes deviate substantially from zero and one, respectively. Such discrepancies between model and realization statistics are referred to as ergodic fluctuations [[13], p. 426]. These fluctuations can be important when the range of the semivariogram model is large with respect to the size of the simulated area, in particular when the nugget effect is small [[31,32] p. 128–133]. In the program **pfield.exe **described below, each set of simulated normal scores is rescaled by its mean and variance to fulfill the underlying assumptions of a standard normal random function.

#### Creating animated displays of realizations

One interesting feature of the p-field simulation approach is its ability to generate easily a series of realizations that are incrementally different. The algorithm, originally proposed by Srivastava [19] for the simulation of raster grids, requires the generation of a probability field that is much larger than the area to be simulated. Multiple realizations are then generated by shifting slightly the probability field before sampling the set of ccdfs. This small shift ensures that, albeit different, the probabilities used to sample the ccdfs from one realization to the next are very close to each other, leading to gradual changes between successive realizations. These realizations can be used as consecutive frames in an animation, enabling a dynamic and evolving display of the uncertainty attached to the spatial distribution of attribute values. A similar approach is here implemented for the simulation of risk values over the non-gridded set of county centroids; see the *Results and Discussion *Section.

### Local cluster analysis and propagation of uncertainty

Once the rates have been geostatistically filtered or simulated, classical Exploratory Spatial Data Analysis (ESDA) techniques can be applied to investigate the existence of local clusters or outliers of high or low cancer risk values. For example, the local Moran test evaluates local clustering or spatial autocorrelation [33]. Its null hypothesis is that there is no association between rates in neighboring geographical units; i.e. counties in this paper. The working (alternative) hypothesis is that spatial clustering exists. For each county, the so-called LISA (Local Indicator of Spatial Autocorrelation) statistic is computed as:



where (**u**_*α*_) is the kriged risk for the county being tested, which is referred to as the "kernel" hereafter; (**u**_*j*_) are the risk values for the *J*(**u**_*α*_) neighboring counties that are here defined as units sharing a common border or vertex with the kernel **u**_*α *_(1-st order queen adjacencies). All values are standardized using the mean *m *and standard deviation *s *of the set of risk estimates. Since the standardized values have zero mean, a negative value for the LISA statistic indicates a negative local auto-correlation and the presence of spatial outlier where the kernel value is much lower (higher) than the surrounding values. Cluster of low (high) values will lead to positive values of the LISA statistic.

In addition to the sign of the LISA statistic, its magnitude informs on the extent to which kernel and neighborhood values differ. To test whether this difference is significant or not, a Monte Carlo simulation is conducted, which traditionally consists of sampling randomly and without replacement the global distribution of rates (i.e. sample histogram) and computing the corresponding simulated neighborhood averages. This operation is repeated many times (e.g. *M *= 999 draws) and these simulated values are multiplied by the kernel value to produce a set of *M *simulated values of the LISA statistic at location **u**_*α *_. This set represents a numerical approximation of the probability distribution of the LISA statistic at **u**_*α *_, under the assumption of spatial independence. The observed statistic (Equation 10) is compared to the probability distribution, enabling the computation of the probability of not rejecting the null hypothesis. The so-called *p*-value is compared to the significance level *α *chosen by the user and representing the probability of rejecting the null hypothesis when it is true (Type I error). Every county where the *p*-value is lower than the significance level is classified as a significant spatial outlier (HL: high value surrounded by low values, and LH: low value surrounded by high values) or cluster (HH: high value surrounded by high values, and LL: low value surrounded by low values). If the *p*-value exceeds the *α *level, the county is declared non-significant (NS).

Instead of conducting the local cluster analysis on the set of smooth risk estimates, one of the key ideas of this paper is to apply the algorithm to the set of simulated risk maps, yielding a set of classifications of counties into significant clusters and outliers, or non significant units. The ensemble of classified maps can be used to compute the probability that a county belongs to each of the five classes. This county is then allocated to the class with the highest probability of occurrence (maximum likelihood classification).

Any prior information on the spatial pattern of mortality risks could be included directly into the randomization procedure underpinning the test of significance of local cluster analysis using the approach proposed by Goovaerts and Jacquez [9]. In other words, the null hypothesis of spatial randomness and uniform risk, corresponding to the operation of randomization of the outcomes, is replaced by models that are more realistic. Realizations of the so-called "Neutral Models" are also generated using geostatistical simulation. The approach allows the identification of spatial patterns above and beyond that incorporated into the neutral model, enabling, for example, the identification of "hot spots" beyond background variation in a pollutant or the detection of clusters beyond regional variation in the risk of developing cancer [34].

### Software

Poisson kriging, including the estimation and modelling of the semivariogram of the risk, was conducted using the public-domain executable **poisson-kriging.exe **described in Goovaerts [8]. The data and parameter file used for the estimation of pancreatic cancer risks are provided with the paper (additional file 1: pancreas.dat, additional file 2: poisson-kriging.par). Probability fields were generated using a modified version of the code **sgsim.exe **from the public-domain Geostatistical Software Library Gslib [32]. This modified code allows simulation over an irregular grid of county centroids, and the random deviates are directly combined with the output of Poisson kriging to generate simulated risk values. The executable of this p-field code is provided with the paper (additional file 3: **pfield.exe**), along with the parameter file used for pancreatic cancer (additional file 4: pfield.par). Local cluster analysis and animation of results were performed using the Space-Time Information System (STIS) technology [24].

**Figure 2:**
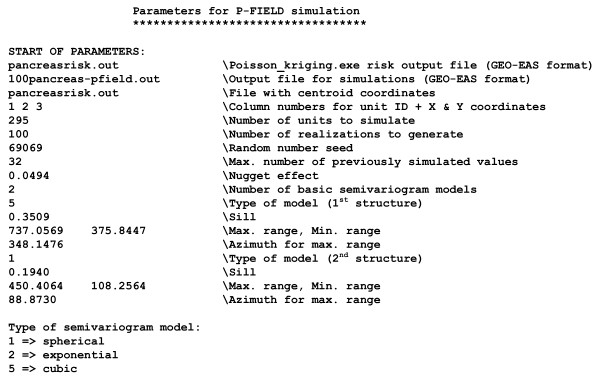
**Example of parameter file required by pfield.exe**. This parameter file is used to generate 100 realizations of the spatial distribution of mortality risk values for pancreatic cancer. The input information includes the risk estimates and variances calculated by Poisson kriging, and the semivariogram of risk fitted by the program **poisson_kriging.exe **and displayed in Figure 5 (right bottom graph).

**Figure 3:**
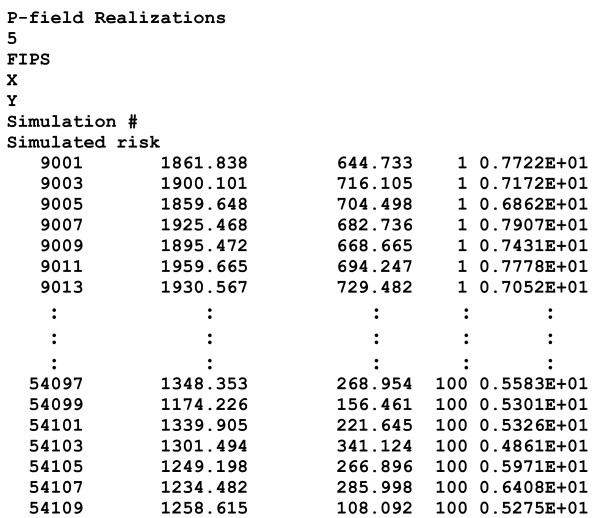
**Output file created by pfield.exe following the analysis of pancreatic cancer mortality rates**. The output file (Geo-EAS format) includes the spatial coordinates of each entity centroid and the simulated values for each realization. This example shows the first and last few lines of the file (i.e. realization 1 to 100).

**Figure 4:**
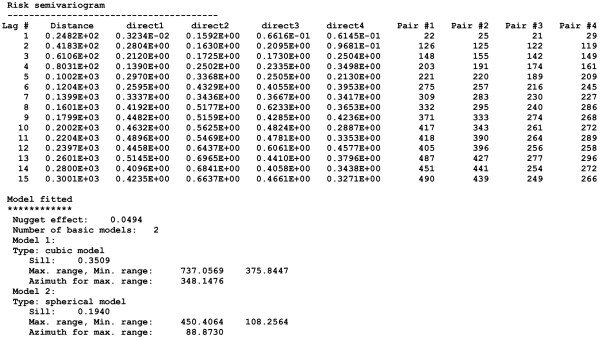
**Output semivariogram file created by poisson_kriging.exe following the analysis of pancreatic cancer mortality rates**. This output file, called **pancreasvariog.txt**, includes the semivariogram risk values computed using 15 classes of 20 km, in four directions with the first direction azimuth starting at 22.5° measured clockwise from the NS axis. This file was created by **poisson_kriging.exe **using the additional files 1 and 2. The parameters of the semivariogram model are copied into the parameter file **pfield.par **required for p-field simulation (Figure 2).

Figure 2 shows the parameter file, **pfield.par**, used to generate 100 realizations of the spatial distribution of pancreatic cancer mortality risk values over 295 county centroids. The text file includes the following information:

• Name of the text file including the Poisson kriging risk estimates and variances. This file is one of the output files of **poisson_kriging.exe.**

• Name of the output text file (Geo-EAS format [35]) that includes the spatial coordinates of each entity centroid and the simulated values for each realization. The output file produced by the parameter file **pfield.par**, **100pancreas-pfield.out**, is shown in Figure 3.

• Name of the text file including the ID field and spatial coordinates of the centroids of the geographical units to be simulated.

• The column numbers for the observation identification code (i.e. FIP county), and the variables with the spatial coordinates.

• The number of geographical units to simulate.

• The number of realizations to generate.

• Random number seed to initiate the random number generator. This number should be a large odd integer.

• The maximum number of previously simulated values to use in the computation of kriging weights and kriging variance (parameter *K *in Equations 7–9). These values are selected according to their covariance with the node being simulated, which accounts for both distance and direction in presence of anisotropy. Values beyond the range of autocorrelation (i.e. zero covariance) are not selected since their weight is systematically zero in simple kriging.

• Parameters of the risk semivariogram model: nugget effect, number and type of basic semivariogram models, sill and range(s) of autocorrelation for each model, azimuth of the direction of maximum range if an anisotropic model is fitted. These values are found in the output text file created by **poisson_kriging.exe**; see example in Figure 4.

## Results and discussion

### Semivariograms of cancer mortality risk

**Figure 5:**
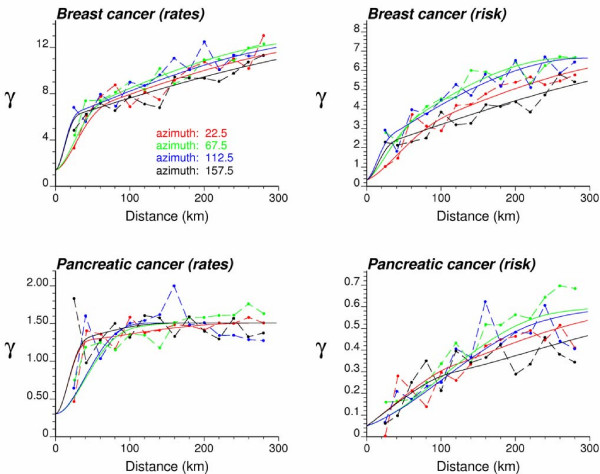
**Directional semivariograms for breast and pancreatic cancer mortality rates and risks with the model fitted**. The semivariograms of raw mortality rates (left column) and the semivariograms of the risk (Equation 4, right column) are computed in four directions; azimuth angles are measured in degrees clockwise from the NS axis. The solid curve denotes the anisotropic (i.e. direction-dependent) model fitted using weighted least-square regression (program **poisson_kriging.exe **in [8]).

The experimental risk semivariograms for breast and pancreatic cancer mortality were estimated using equation (4) along four directions. Figure 5 (right column) shows the results, while the left column shows the traditional semivariograms computed directly from the observed rates. On each graph, the solid curve denotes the model fitted using weighted least-square regression. For example, the traditional semivariogram estimator for pancreatic cancer was modeled using two cubic models: (min. range = 50 km, max. range = 450 km) and (min. range = 158 km, max. range = 332 km). The risk semivariogram was modeled using a combination of a spherical model (min. range = 108 km, max. range = 450 km) and cubic model (min. range = 376 km, max. range = 737 km).

The semivariograms of the risk are much better structured and have smaller sills than the corresponding semivariograms of the rates. This is expected since the weights in expression (4) attenuate the influence of extreme rates computed from small population sizes and subtraction of the correction term m* reduces the variance even more. This effect is more pronounced for pancreatic cancer: the relative decrease in sill value is larger than for breast cancer, and the risk semivariogram has a much longer range of autocorrelation than the traditional estimator that is almost pure nugget effect (i.e. no spatial correlation). Since pancreatic cancer is less frequent than breast cancer, its mortality rates are more likely to be impacted by the small number problem and display higher levels of noise. In particular for breast cancer the semivariogram of the risk displays a slight anisotropy, with smaller variability (red curve) along the NE-SW direction for small distances.

### Poisson kriging

**Figure 6:**
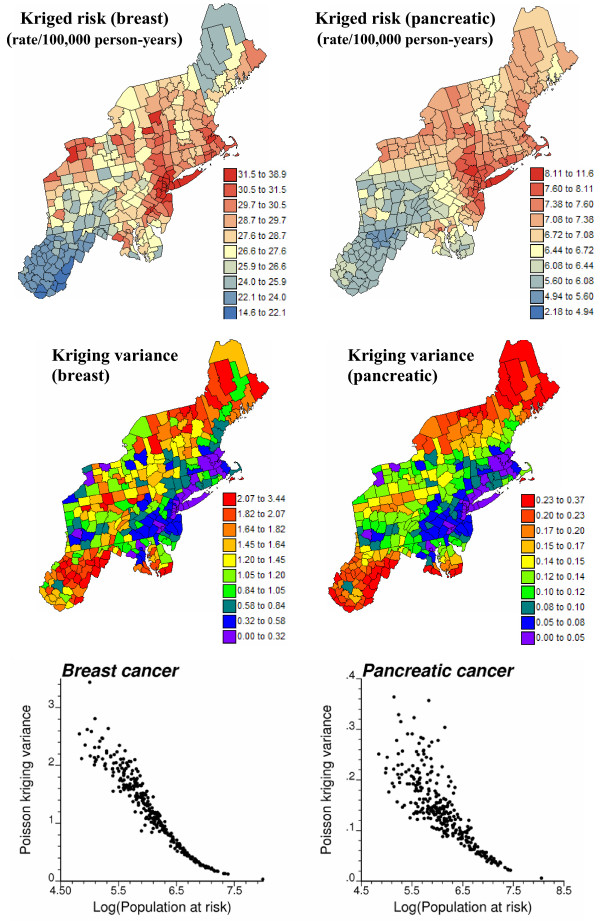
**Maps of breast and pancreatic cancer mortality risks estimated by Poisson kriging and the corresponding kriging variance**. For the two top maps, the fill color in each county represents the cancer mortality risk per 100,000 person-years estimated for the period 1970–1994; the class boundaries correspond to the deciles of the histogram of rates. The middle maps display the kriging variance computed using Equation (2); the class boundaries correspond to the deciles of the histogram of kriging variances. Bottom scattergrams illustrate the greater uncertainty of the risk estimated for sparsely populated counties.

**Figure 7:**
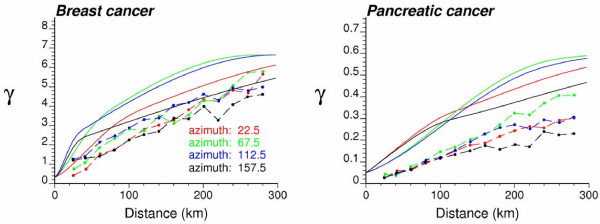
**Reproduction of the risk semivariogram model by the set of risk estimates**. The semivariogram of risk estimates is computed in four directions; azimuth angles are measured in degrees clockwise from the NS axis. The solid curve denotes the anisotropic (i.e. direction-dependent) model used in Poisson kriging. This graph illustrates the underestimation of the spatial variability of the risk by the smooth map of kriging estimates.

Risk semivariogram models were used to estimate the cancer mortality risk and the associated prediction variance by Poisson kriging. Following a previous study [8], the closest 32 neighboring counties were used for the estimation. The risk maps in top of Figure 6 are much smoother than the original rate maps (Figure 1), since the noise caused by small population sizes has been filtered. This smoothing effect is also reflected by the lack of reproduction of the risk semivariogram model by the set of kriging estimates, see Figure 7. Especially for pancreatic cancer, there is a severe underestimation of the short-range variability, which is a typical feature of kriging and other least-square interpolation algorithms [[13], p. 372]. These maps, however, facilitate the visualization of areas of higher mortality risks, such as the East coast around the cities of New York and Boston, while West Virginia and Southern Pennsylvania have much lower risks for both cancers. The two risk maps mainly differ in the Northern part of the study area, with higher risks for pancreatic cancer in Maine. Yet, these predictions are based on sparsely populated or geographically remote counties, hence the associated kriging variance is very large; see Figure 6 (middle maps). Large kriging variances are also found in the South where mortality risks for both cancers are lower than average. The bottom scattergrams in Figure 6 illustrate the relationship between the kriging variance and the population size, indicating the expected higher reliability of risk values estimated from densely populated counties.

**Figure 8:**
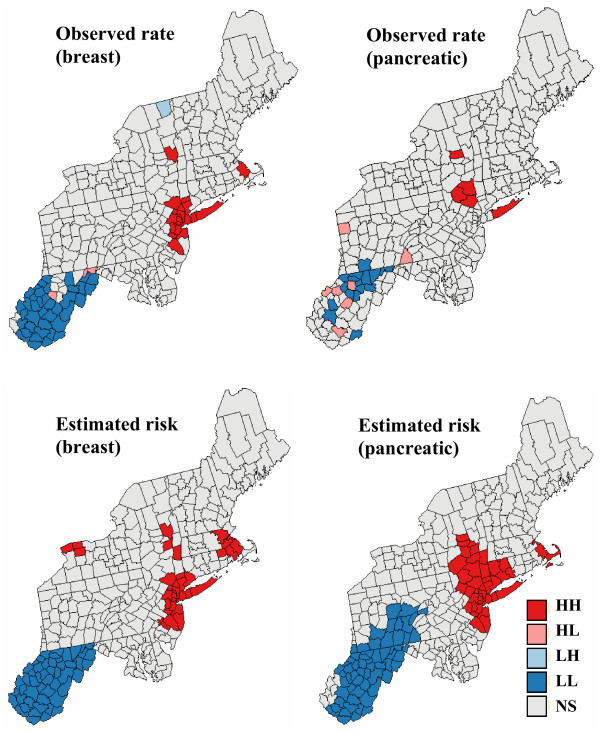
**Results of a local cluster analysis conducted on breast and pancreatic cancer mortality rates and estimated risks**. The fill color in each county represents the classification into significant low-low (LL) or high-high (HH) clusters, as well as high-low (HL) or low-high (LH) outliers. Light gray indicates counties that are not significant at the level *α *= 0.05; the *p*-values were corrected for multiple testing using the Simes adjustment.

Aggregates of counties with lower or higher mortality risks are easily detected in the local cluster analysis, whose results are displayed in Figure 8 (bottom graphs). A significance level *α *= 0.05 was used and the *p*-values were corrected for multiple testing using the Simes adjustment [36]. The analysis of risk estimates indicates significant low-low clusters (LL) in West Virginia, and part of Pennsylvania for pancreatic cancer. Significant clusters of high risks (HH) are found around New York and Boston for both cancers.

The comparison of results obtained for the observed mortality rates versus risk estimates illustrates the impact of noise filtering on the local cluster analysis. For both cancers, the few spatial outliers detected for mortality rates are not significant in terms of risk. For example, the only significant low-high outlier for breast cancer, Clinton County (NY), was caused by the very large and unreliable age-adjusted breast cancer mortality rate of 38.8 per 100,000 person-years observed in its eastern neighbor, the small county of Grand Isle (VT); see Figure 8 (left top graph). The estimated risk in this sparsely populated county drops to 28.4, leading to a much smaller neighborhood value and the disappearance of the low-high outlier. Another consequence of the smoothing is the expansion of the clusters' size. Table 1 indicates that 50% to 400% more counties are classified as significant clusters based on risk maps versus the original mortality rates. For breast cancer, the cluster of low risks in the South now includes the two high-low outliers detected on the map of mortality rates. Note also in the western part of the map the high-high cluster of three New-York counties, including Niagara, which appears after application of Poisson kriging. This result agrees with the identification of a breast cancer cluster in the Western New-York area by the New-York State's Department of Health [37]. Differences between the classifications of mortality rates and risks are the most important for the less frequent pancreatic cancer, since the rates are expected to be less reliable; see Figure 8 (right column). The LL and HH clusters expanded into much larger clusters on the classified risk maps, while all seven spatial outliers were smoothed out.

**Table 1:** Results of local cluster analysis of mortality rate and risk maps. Number of counties classified as significant spatial clusters or outliers for breast and pancreatic cancers on the original mortality rate maps, kriged risk maps, and on average over the 500 simulated risk maps.

Maps	HH cluster	LL cluster	HL Outlier	LH outlier
Breast cancer				
Mortality rates	20	39	2	1
Risk estimates	35	50	0	0
Simulation	37.2	48.2	0.18	0.07
				
Pancreatic cancer				
Mortality rates	5	16	7	0
Risk estimates	42	60	0	0
Simulation	44.5	51.0	0.24	0.08

### Stochastic simulation of risk values

A shortcoming of the local cluster analysis in Figure 8 is that it ignores the uncertainty attached to the predicted risks. For example, the large cluster of low mortality risk for pancreatic cancer should be interpreted cautiously because of the large prediction variance in the Southern part of the study area. One notices also the absence of spatial outliers for both cancers which, to some extent, results from the smoothing of high and low values by kriging. This conditional bias (i.e. overestimation of low values and underestimation of high values) might cause some counties to be wrongly classified as non-significant too.

**Figure 9:**
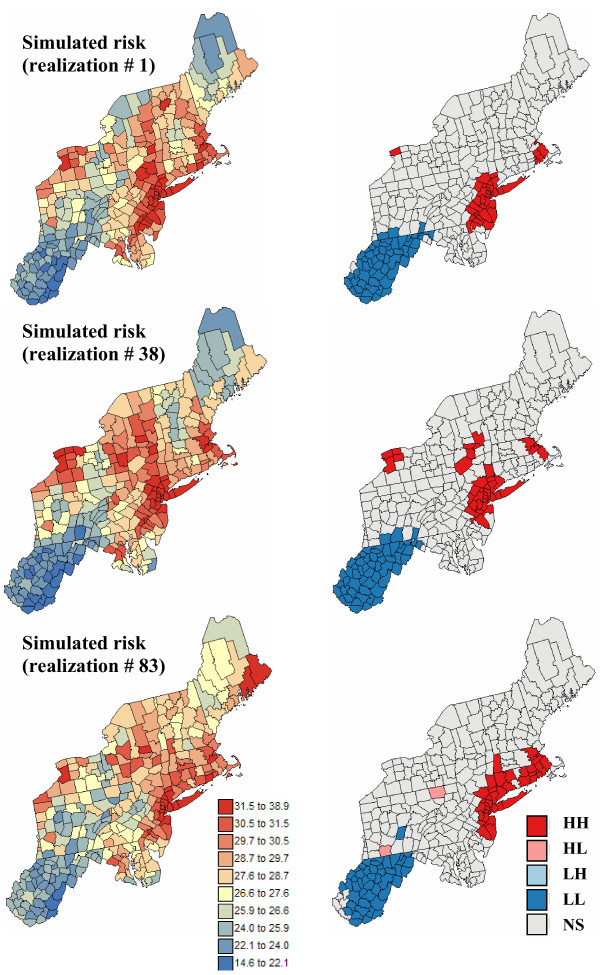
**Simulated risk maps for breast cancer, and results of the local cluster analysis**. For the risk maps (left column), the fill color in each county represents the breast cancer mortality risk per 100,000 person-years simulated for the period 1970–1994; the class boundaries correspond to the deciles of the histogram of rates. For the local cluster analysis (right maps), the fill color in each county represents the classification into significant low-low (LL) or high-high (HH) clusters, as well as high-low (HL) or low-high (LH) outliers. Light gray indicates counties that are not significant at the level *α *= 0.05; the *p*-values were corrected for multiple testing using the Simes adjustment.

**Figure 10:**
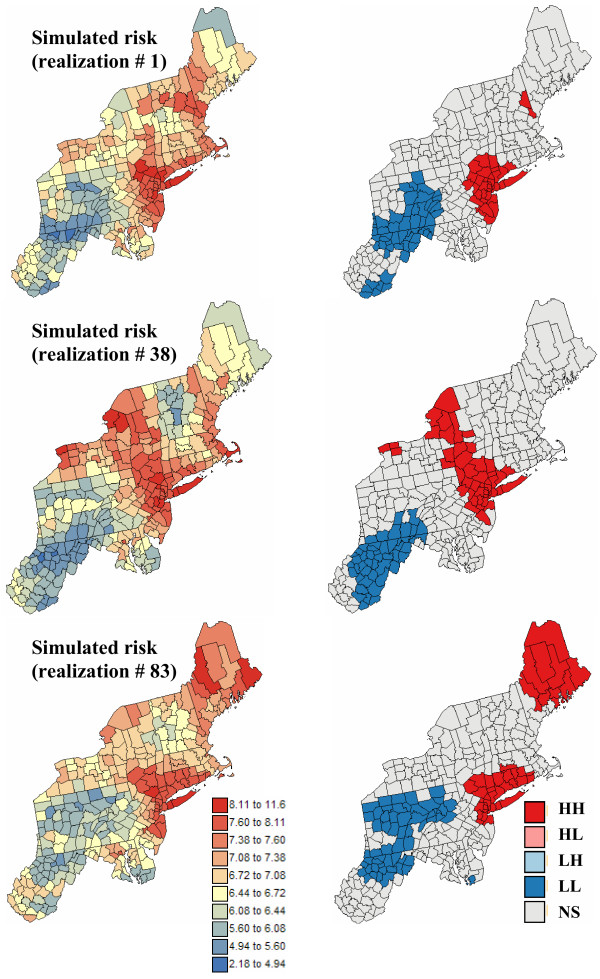
**Simulated risk maps for pancreatic cancer, and results of the local cluster analysis**. For the risk maps (left column), the fill color in each county represents the pancreatic cancer mortality risk per 100,000 person-years simulated for the period 1970–1994; the class boundaries correspond to the deciles of the histogram of rates. For the local cluster analysis (right maps), the fill color in each county represents the classification into significant low-low (LL) or high-high (HH) clusters, as well as high-low (HL) or low-high (LH) outliers. Light gray indicates counties that are not significant at the level *α *= 0.05; the *p*-values were corrected for multiple testing using the Simes adjustment.

Five hundreds realizations of the spatial distribution of risk values were simulated using the approach outlined in the *Methods *Section. Figures 9 and 10 show three realizations and the results of the corresponding local cluster analysis. The simulated maps are more variable than the kriged risk maps of Figure 6, yet they are smoother than the maps of potentially unreliable rates of Figure 1. Differences among realizations depict the uncertainty attached to the risk maps. For example, in Maine and Northwestern part of New-York state the simulated risk for pancreatic cancer takes a wide range of values, emphasizing the lack of reliability of high risk estimates in these regions. Differences between realizations are much smaller for breast cancer, which is expected given the higher frequency of this disease leading to more reliable risk estimates.

**Figure 11:**
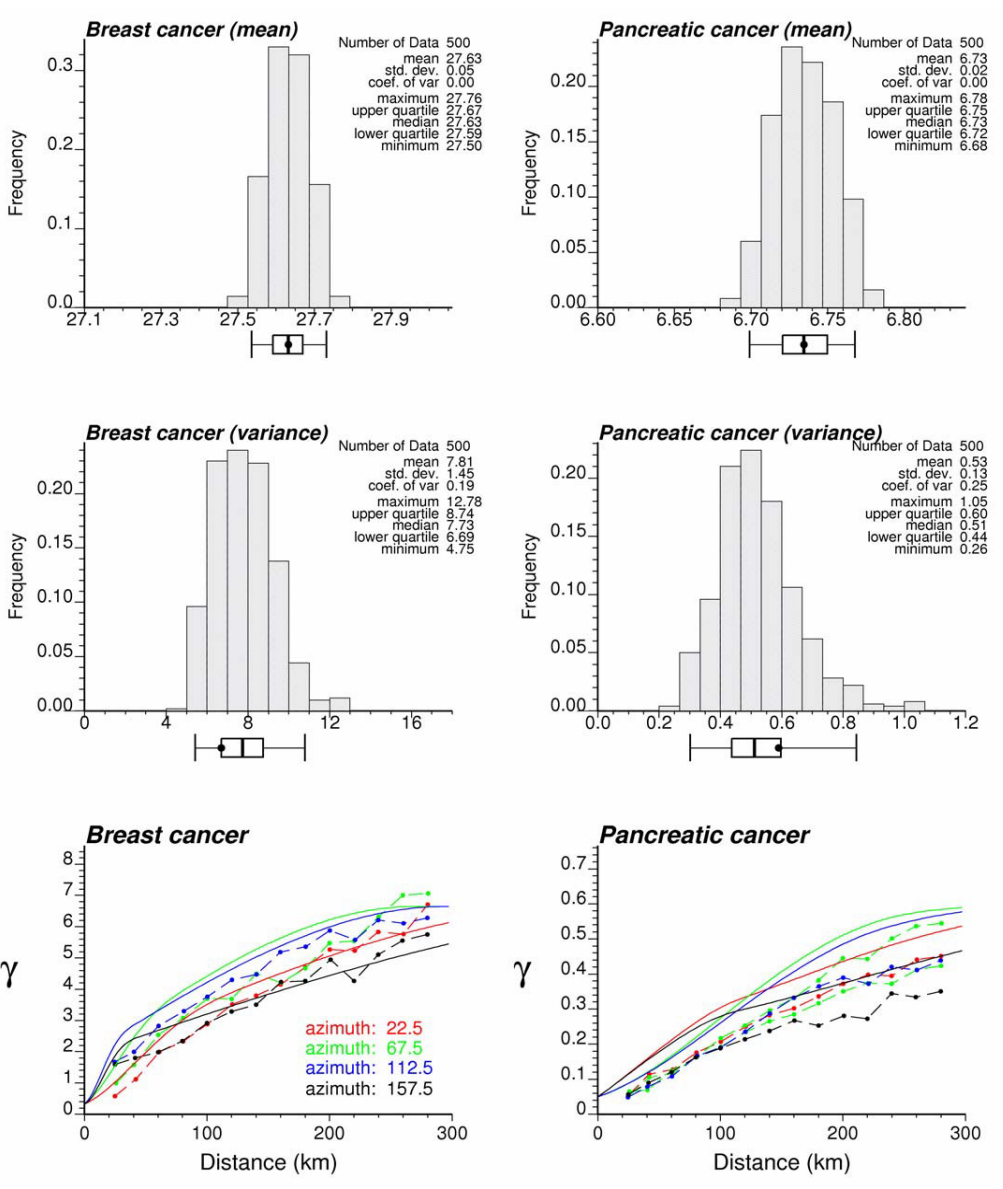
**Reproduction of target statistics by the set of simulated risk maps**. The top and middle graphs show the histograms of the mean and *a priori *variance of each set of simulated values. The black dot in the box plot below each histogram is the target value identified as the average estimated risk or the sill of the risk semivariogram model. Five vertical lines are the 0.025 quantile, lower quartile, median, upper quartile, and 0.975 quantile of the distribution. Bottom graphs show the directional semivariograms of simulated risk values, averaged over all 500 realizations; the solid line depicts the target model fitted in Figure 5.

Reproduction of a target histogram and semivariogram is a common way for the geostatistician to assess the quality of a simulated map [14,18]. Despite the lack of risk data to compute a target histogram, target summary statistics, such as mean and *a priori *variance [38], can be identified with the mean of the risk estimates and the sill of the risk semivariogram model, respectively. Figure 11 shows the histogram of these two statistics computed from the set of 500 simulated maps. The *a priori *variance corresponds to the sill of the model fitted to the directional semivariograms computed for each set of simulated risk values; this statistic is a better measure of dispersion than the sample variance since it accounts for the spatial correlation between risk values (i.e. variance of data separated by a distance larger than the range of autocorrelation). The black dot in the box plot below each histogram is the target value, i.e. the average estimated risk or the sill of the risk semivariogram model. Five vertical lines are the 0.025 quantile, lower quartile, median, upper quartile, and 0.975 quantile of the distribution. The simulated maps reproduce, on average, reasonably well the two target statistics, in particular the target mean. The coefficient of variation is smaller for breast cancer relative to pancreatic cancer. Besides the reproduction of the sill, reproduction of the shape of the target semivariogram model should also be assessed. The directional semivariograms of simulated risk values were computed for each of the 500 realizations, and their average is superimposed on the target model (solid curve) at the bottom of Figure 11. For both cancers the simulated risk maps very well reproduce the spatial anisotropy; the sill is slightly underestimated in the realizations of pancreatic mortality risks, which agrees with the underestimation of the target *a priori *variance noticed on the histogram. Discrepancies between the realization and target semivariograms (i.e. ergodic fluctuations) in this application are, to a large extent, explained by the large range of autocorrelation (up to 800 km) with respect of the size of the study area, as well as the small nugget effect of the risk semivariogram models. Yet, comparison of Figures 11 and 7 shows that simulated maps better reproduce the spatial variability of risk values, as modeled by the semivariogram function *γ*_*R*_(**h**), than the corresponding kriged maps.

**Figure 12:**
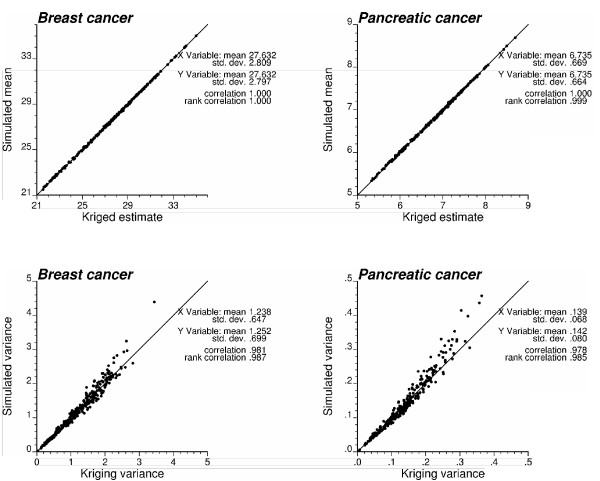
**Reproduction of Poisson kriging estimate and variance by the set of simulated risk values**. By construction, the mean and variance of the local (i.e. county-specific) distribution of simulated risk values should be equal to the mean (kriging estimate) and variance (kriging variance) of the ccdf sampled using the probability field. The scatterplots indicate that the ensemble of 500 realizations provides a uniform sampling of the local distributions of probability.

By construction, the mean and variance of the local (i.e. county-specific) distribution of simulated risk values should reproduce the mean and variance of the ccdf that is repeatedly sampled across all realizations. The ccdf parameters are the kriging estimate and variance, and those are plotted against the mean and variance of 500 simulated risk values in Figure 12. The good agreement between the two sets of statistics, in particular the mean, indicates that the ensemble of 500 probability fields provides a uniform sampling of these local distributions of probability.

### Propagation of uncertainty through local cluster analysis

The classified maps in the right column of Figures 9 and 10 illustrate how the uncertainty about risk values translates into uncertainty about the results of the local cluster analysis. From one realization to another, the shape and position of local clusters can change substantially. For example, the southern part of the cluster of low risk values detected on the kriged map for pancreatic cancer becomes non significant on some realizations; see Figure 10 (bottom right graph). The location and nature of clusters is much more stable for breast cancer. Table 1 indicates that slightly more counties are classified as significant clusters when the analysis is performed on kriged risks instead of simulated values. A few counties are also now classified as significant outliers, which confirms the potential bias of an analysis based on smoothed rates.

**Figure 13:**
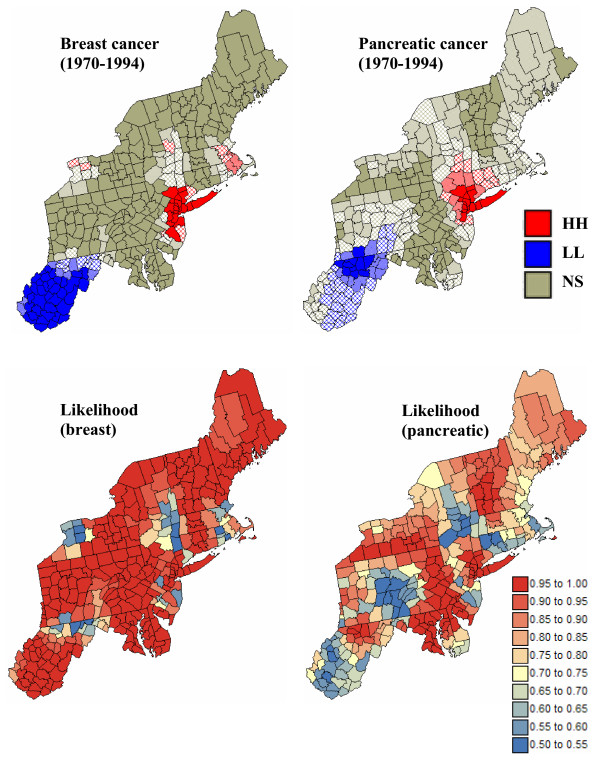
**Summary of local cluster analyses conducted on 500 simulated risk maps**. For the top maps, the fill color in each county represents the most frequent classification observed over 500 simulations of the spatial distribution of breast and pancreatic cancer mortality risks: low-low (LL) or high-high (HH) clusters, while light gray indicates counties that have mostly been found non-significant at an *α *level of 0.05; the *p*-values were corrected for multiple testing using the Simes adjustment. The intensity of the shading increases as the classification becomes more certain; the classification likelihood is mapped in the bottom maps.

The information provided by the set of 500 local cluster analyses is summarized in Figure 13. The color code in the top maps indicates the most frequent classification of each county across the 500 simulated maps. The shading reflects the probability of occurrence or likelihood of the mapped class. Solid shading corresponds to classifications with high frequencies of occurrence (i.e. likelihood > 0.9), while hatched counties denote the least reliable results (i.e. likelihood < 0.75). This coding is somewhat subjective but leads to a clear visualization of the lower reliability of the classification obtained for pancreatic cancer versus breast cancer; a similar conclusion can be drawn from the probability maps displayed at the bottom of Figure 13. The classification likelihood, averaged over all 295 counties, is 0.92 for breast cancer versus 0.81 for pancreatic cancer. In particular, the classification as low risk areas for pancreatic cancer exceeds a 0.9 likelihood only for a few counties in the large cluster detected in West Virginia and Southern Pennsylvania on the map of Figure 8 (right bottom). As intuitively expected, less reliable results are found for counties located on the edge of the clusters as well as for isolated counties. It is noteworthy that Niagara county is still classified as a high-high cluster with a 0.6 probability of occurrence. None of the county is predominantly classified as outlier across all realizations.

### How many realizations are needed?

**Figure 14:**
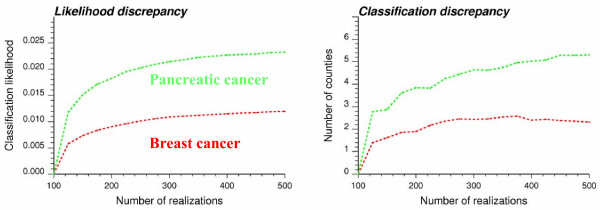
**Impact of the number of realizations on the stability of local cluster analysis results**. The left graph displays the absolute change in classification likelihood as the number of simulated risk maps increases from 100 to 500. The right graph shows the number of counties that are classified differently as the number of realizations increases from 100 to 500. Both curves represent results averaged over 50 random subsets.

The use of stochastic simulation in test of hypothesis relies on the assumption that the space of solution is sampled fairly exhaustively and uniformly (equally-probable realizations [18]). It is thus necessary to investigate how conclusions change as a function of the number of simulated maps generated. For example, Figure 14 shows the influence of increasing the number of realizations from 100 to 500 on the average difference in terms of likelihood values and classification of counties into significant outliers and clusters (the reference is the results obtained using 100 realizations). To reduce sampling fluctuations, for each sample size ranging from 100 to 500 realizations, 50 subsets were randomly selected from the original set of 500 realizations, and the averaged results are plotted in Figure 14. All curves exhibit a plateau within this range of number of realizations, although the asymptotic behavior depends on the type of cancer. Larger classification discrepancies and slower convergence are observed for pancreatic cancer, which confirms previous results regarding its lower classification reliability relative to breast cancer. Yet, for this case study, enough realizations of the spatial distribution of cancer mortality risks were generated to yield a stable classification of counties.

### Visualization of spatial uncertainty

**Figure 15:**
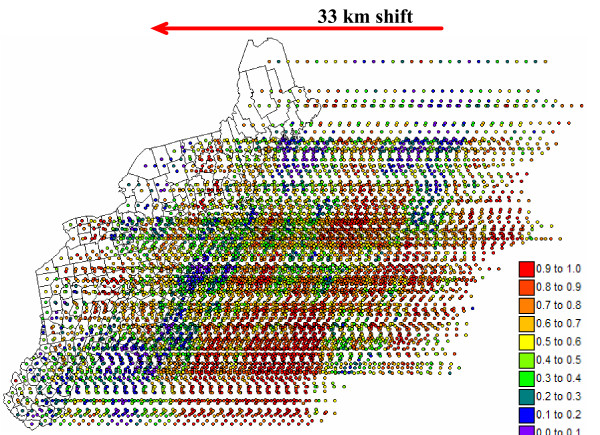
**Illustration of the p-field approach for generating animated displays of simulated maps. **A large simulation grid is first generated by shifting the 295 county centroids by EW increments of 33 km. This large grid is then populated with probability values using sequential Gaussian simulation and the risk semivariogram models of Figure 5. Last, the probability values are used to sample Gaussian local distributions of probability characterized by the Poisson kriging estimate and variance mapped in Figure 6 (Monte-Carlo simulation).

**Figure 16:**
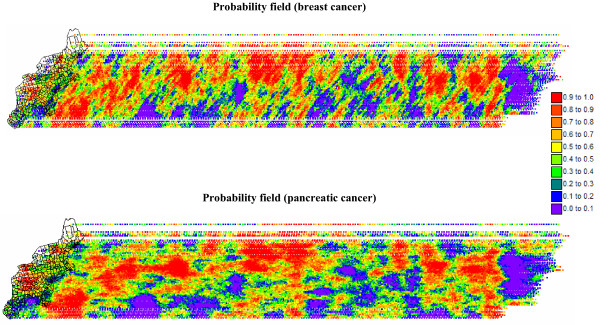
**Probability fields used to create incrementally different simulated maps of breast and pancreatic cancer mortality risk**. These large probability fields were generated by sequential Gaussian simulation and used to create 200 realizations of simulated risk values according to the procedure described in Figure 15.

A series of 200 incrementally different simulated risk maps was generated by shifting a very large probability field by an EW increment of 33 km between realizations; see an example in Figure 15 for breast cancer. 33 km is the average distance between centroids of contiguous counties. East West corresponds to the approximate direction of largest variability displayed by the risk semivariograms, and it is expected to lead to a more complete exploration of the space of uncertainty through the maximization of differences among realizations. Figure 16 shows the entire probability fields used for both cancers. The breast cancer probability field shows clear NE-SW bands of high and low probabilities, which reflects the anisotropy displayed by the risk semivariogram model in Figure 5. The anisotropy is much less pronounced for pancreatic cancer. Note that since the same random numbers and random path were used in sequential simulation for both cancers, the locations of high and low values are fairly similar in the two probability fields.

**Figure 17:**
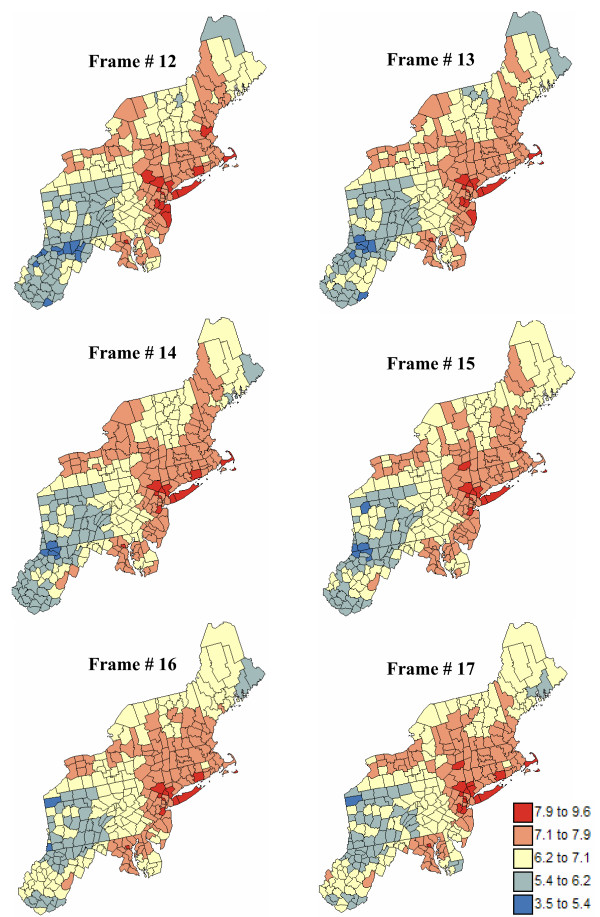
**Six consecutive frames of the animated display of mortality risk maps for pancreatic cancer**. The fill color in each county represents the pancreatic cancer mortality risk per 100,000 person-years simulated for the period 1970–1994; fewer class boundaries are selected to enhance the spatial features in all maps.

**Figure 18:**
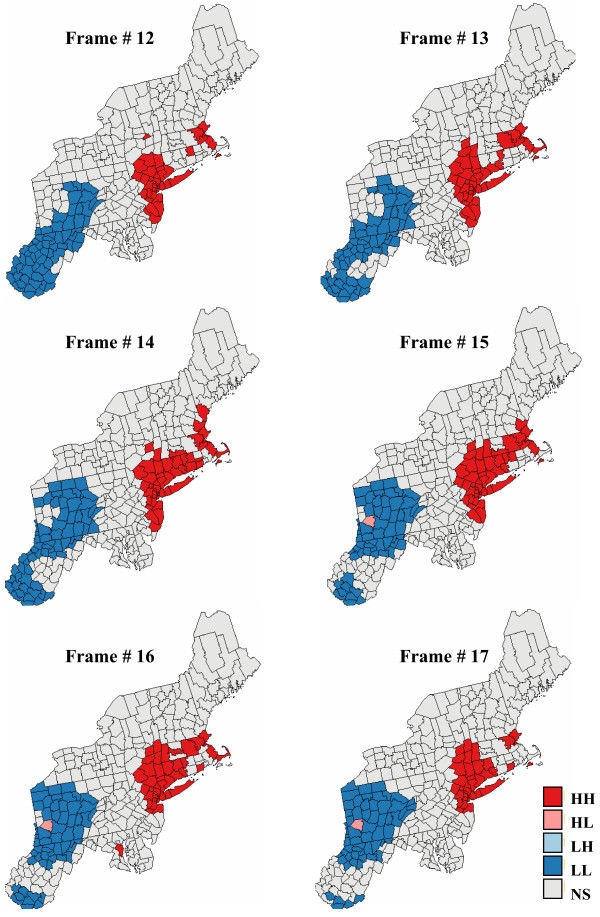
**Six consecutive frames of the animated display of local cluster analysis results for pancreatic cancer**. The fill color in each county represents the classification into significant low-low (LL) or high-high (HH) clusters, as well as high-low (HL) or low-high (LH) outliers. Light gray indicates counties that are not significant at the level *α *= 0.05; the *p*-values were corrected for multiple testing using the Simes adjustment. The six maps are obtained by performing a local cluster analysis of the six maps of simulated pancreatic cancer mortality risks displayed in Figure 17.

Figure 17 shows six consecutive realizations of the animated set created for pancreatic cancer mortality risk; the entire animation is available as additional file 5: pancreas.avi. The most striking feature is the gradual increase of risk values in West Virginia, while risk values decrease in the Southeastern part of New Jersey. This gradual change in spatial pattern of risk values between successive frames of the animation leads to a gradual change in the position and nature of clusters and outliers. Figure 18 depicts a shrinking of the low-low cluster in the South, while the same cluster is expanding to the North. This change illustrates the lack of reliability of the classification of these counties as low-low cluster on the map of Figure 8 (right bottom graph), a feature captured by the summary map of Figure 13 (right top graph). Similarly, a few counties in the Southeastern part of New Jersey classified as high-high cluster on Frame # 12 become non-significant on Frame #17. The entire animation of local cluster results is available as additional file 6: pancreas-LCA.avi.

## Conclusion

Capitalizing on the abundant geostatistical literature devoted to the modelling of local and spatial uncertainty plus the recent development of Poisson kriging, this paper presented a novel approach, and the corresponding computer code, to generate realizations of the spatial distribution of risk values. P-field simulation proceeds in two steps. First, the local uncertainty about the risk prevailing within each geographical unit is modelled from the observed mortality rates and population at risk using Poisson kriging. The set of local probability distributions is then sampled using a set of spatially correlated probability values generated using non-conditional sequential Gaussian simulation. Through sampling by slowly moving probability fields, the approach also allows the creation of incrementally different realizations that can be used as consecutive frames in an animation, enabling a dynamic and evolving display of the uncertainty attached to the spatial distribution of risk values.

Simulated risk maps reproduce the spatial variability of the risk, thereby overcoming the smoothing effect inherent to all current procedures for stabilization of rate data, including Poisson kriging. In fact, the map of kriged risk is but the average of all simulated risk maps, while the kriging variance corresponds to the variance of the local distribution of simulated risk values. The probability of occurrence of multi-point or spatial features, such as aggregates of counties with low or high cancer mortality risk, can also be assessed numerically from the ensemble of realizations. This information can be conveyed through static maps of spatial clusters and outliers, using a color code indicating the likelihood of the classification, or through the dynamic display of the set of classified maps.

The analysis of breast and pancreatic cancer mortality rates illustrated the potentialities of the approach for modelling and propagating their spatial uncertainty through local cluster analysis. Geostatistical simulation generated risk maps that are more variable than the smooth risk map estimated by Poisson kriging and reproduce better the spatial pattern captured by the risk semivariogram model. Differences between realizations were particularly important for the less frequent pancreatic cancer, reflecting for example the uncertainty attached to higher risk estimates in sparsely populated counties of Maine and North-western New-York. Such uncertainty translates into a lack of reliability of some of the spatial clusters and outliers detected on the map of smoothed rates. The case study showed how geostatistical simulation permits the quantification of the likelihood of spatial clusters and outliers detected using Moran's I. Local cluster analysis of the set of simulated risk maps leads to a clear visualization of the lower reliability of the classification for pancreatic cancer versus breast cancer: only a few counties in the large cluster of low risk detected in West Virginia and Southern Pennsylvania are significant over 90% of all simulations. On the other hand, the cluster of high breast cancer mortality in Niagara county, detected after application of Poisson kriging, is significant for 60% of simulated risk maps. Sensitivity analysis shows that 500 realizations are needed to achieve a stable classification for pancreatic cancer, while convergence is reached after fewer than 300 realizations for breast cancer.

Maps of cancer incidence as well as mortality rates are frequently used as input to disease clustering procedures whose purpose is to identify local areas of excess and deficit. Most users recognize that rates recorded for small populations are uncertain, leading to the routine application of smoothing methods prior to the analysis. Yet, in their interpretation of the risk maps, they tend to forget that the risks themselves are prone to uncertainty and that some features, such as large clusters of low or high risk, might just be artifacts of the smoothing method. Ignoring such uncertainty can lead to misallocation of resources to investigate unreliable clusters of high risk, while areas of real concern might go undetected. Instead of a single, and potentially misleading, map of smoothed rates, the simulation approach provides public health officials with a set of plausible scenarios for the spatial pattern of risk. Those permit investigating the impact of risk uncertainty on alternate intervention and control activities. In the future, the concept of stochastic simulation of mortality risks will be combined with the neutral model methodology [9] to assess geographic clustering using appropriate null hypotheses that account for the spatial correlation and background variation modeled from the observed rates and any ancillary information (i.e. exposure model).

## Competing interests

The author is affiliated with BioMedware a research company that also develops software for the exploratory spatial and temporal analysis of health and environmental data. With funding from the National Cancer Institute, the author developed STIS (Space-Time Intelligence System), which is a commercial product of Terraseer and should include Poisson kriging and stochastic simulation functions in a future release.

## Supplementary Material

Additional File 1 Input data file for poisson_kriging.exe. This dataset follows the Geo-EAS format and includes, for 295 counties of New England, the following information: FIPS code, spatial coordinates of the county geographic centroid, age-adjusted pancreatic cancer mortality rate per 100,000 person-years, and the population at risk for the 1970–1994 period. Click here for file

Additional File 2 Input parameter file for poisson_kriging.exe. This text file includes all the variables and names of input/output files required by the program, as well as the parameters for semivariogram modelling and Poisson kriging. Click here for file

Additional File 3 Executable to generate realizations of the spatial distribution of risk values using p-field simulation. Click here for file

Additional File 4 Input parameter file for pfield.exe. This text file includes all the variables and names of input/output files required by the program, as well as the parameters for the simulation procedure. Click here for file

Additional File 5 Animated display of 200 realizations of the spatial distribution of mortality risk values for pancreatic cancer. Click here for file

Additional File 6 Animated display of 200 realizations of the local cluster analysis of mortality risk values for pancreatic cancer. Click here for file
